# Effects of mental disorders on the relationship between physical activity and bone markers among depressed patients in Germany

**DOI:** 10.1016/j.pmedr.2025.103364

**Published:** 2025-12-27

**Authors:** Sanne Houtenbos, Yangyang He, Andrea Block, Pia-Maria Wippert

**Affiliations:** a Medical Sociology and Psychobiology, Department of Health and Physical Activity, University of Potsdam, 14469 Potsdam, Germany; b Faculty of Health Sciences Brandenburg, Joint faculty of the University of Potsdam, the Brandenburg, Medical School Theodor Fontane and the Brandenburg University of Technology Cottbus—Senftenberg, 14469 Potsdam, Germany

**Keywords:** Osteoporosis, Depressive symptoms, Bone disorders, Interaction effects, Psychosomatic symptoms

## Abstract

**Objective:**

Physical activity (PHYA) positively influences bone health, but this relationship might be affected by mental disorders. Aims: (1) evaluate whether mental disorders influence bone markers and (2) whether the relationship of PHYA with bone markers is influenced by mental disorders.

**Methods:**

A secondary data analysis of the longitudinal DEPREHA-study (Germany, 18 months, *n* = 208, 18-65y, ICD-10 F32.x-F33.x, collected 2015–2017) was conducted. Baseline data: sociodemographic, PHYA, depressive (BDI-II) and psychosomatic (SCL-90) symptoms; bone markers procollagen-type-1-N-propeptide (P1NP), osteocalcin (OC) and crosslaps (CTx) were analyzed. Multiple linear regressions assessed associations of depressive and psychosomatic symptoms with bone markers and moderating effects of depressive and psychosomatic symptoms on the PHYA-bone marker relationship; Mediation analyses were conducted with the PROCESS macro. All analyses were conducted in IBM SPSS (v31.0; *p* < 0.05).

**Results:**

Data from *n* = 44 depressed participants were eligible for analysis. Significant associations of BDI-II and SCL-90 with P1NP and OC were detected. A significant moderation of SCL-90 on the relationship of PHYA with P1NP (*p* < 0.05), but no mediation effect of BDI-II or SCL90 on bone markers was detected.

**Conclusions:**

Depressive and psychosomatic symptoms influence and may potentially moderate the relationship between PHYA and bone markers, which should be considered in further studies and therapeutic planning.

## Highlights

• First paper on the role of mental disorders in the physical activity-bone link
• Depressive and psychosomatic symptoms influence bone markers
• Mental disorders may moderate the relationship between physical activity and bone

## 1 Introduction

Osteoporosis is a prevalent bone disorder characterized by reduced bone mineral density (BMD), which increases the risk of fractures and injuries. (Pouresmaeili et al., 2018; Clynes et al., 2020). According to the study ‘Gesundheit in Deutschland Aktuell’ (GEDA 2014/2015), 5.0 % of the German population noted that they had a diagnosis of osteoporosis in the last 12 months (Fuchs et al., 2017). Within the European Union, approximately 22 million women and 5.5 million men were estimated to have osteoporosis in 2010, with 3.5 million new fragility fractures (Hernlund et al., 2013). Bone metabolism is regulated by various mechanisms, including stem cells, osteoblasts (anabolic) and osteoclasts (catabolic) (Kim et al., 2020), and commonly measured through dual-energy X-ray absorptiometry (DEXA) scans, which is the gold standard for BMD measurement. However, bone turnover markers, such as pro-collagen peptides (e.g. P1NP), crosslaps (CTx), osteocalcin (OC), have gained popularity as biomarkers for bone metabolism and treatment of bone pathologies (Wheater et al., 2013).

Frequent risk factors for osteoporosis include aging, a sedentary lifestyle, lack of nutritious foods, smoking, genetic factors, metabolic disorders, and a history of mental disorders (Pouresmaeili et al., 2018; Avgerinou et al., 2024). Mental disorders, such as depression and somatic symptoms (National Collaborating Centre for Mental Health UK, 2011), can affect bone health through various pathways. One perspective represents the behavioral pathway that people who suffer of mental disorders commonly portray different lifestyle choices, such as limited physical activity (PHYA), which could influence bone metabolism (Rosenblat et al., 2016). Alternatively, biological factors, including allosteric processes, represent another pathway in which mental disorders influence bone health (Rosenblat et al., 2016; Guidi et al., 2020). A constant or chronic high stress load, linked to mental disorders, can lead to dysregulation of the hypothalamic-pituitary-adrenal (HPA)-axis, which is followed by various metabolic and endocrine processes, including changes in growth hormones, inflammatory cytokines, glucocorticoids, oxidative and nitrosative stress, and molecular markers (e.g. miRNAs in extracellular vesicles), which all affect bone metabolism (Rosenblat et al., 2016; Guidi et al., 2020; He et al., 2021). Yet, research on the effects of symptomology associated with mental disorders on bone health is limited.

**Fig. 1:**
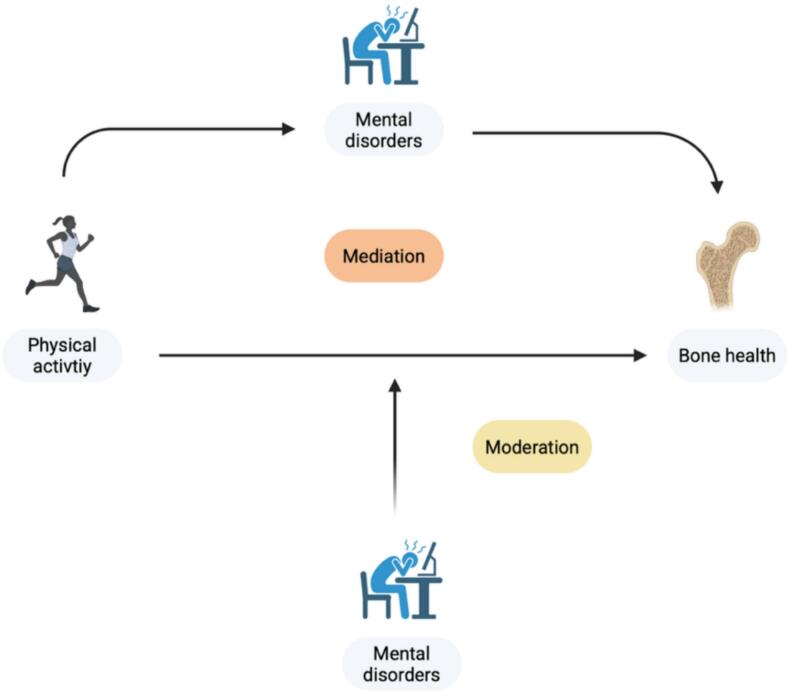
Possible mediation and moderation pathways of mental disorders on the relationship between physical activity and bone health.

Even though mental and bone disorders are separate diseases, they commonly occur simultaneously among patients (Kashfi et al., 2022; Chen et al., 2024; Puth et al., 2018). Treatment of bone pathologies commonly consists of pharmacological treatment, including antiresorptive and osteoanabolic therapies, which come with possible negative side effects (Foessl et al., 2023). PHYA offers an alternative treatment form to improve bone health, having shown improved bone density at the forearm and reduced bone loss at the femoral neck and lumbar spine among people participating in resistance and multicomponent training programs (Gregson et al., 2022). Nonetheless, PHYA treatment forms for bone health generally don't consider the crosstalk between mental disorders and bone pathologies. On the one hand, mental disorders could prove to be potential mediators of the relationship of PHYA with bone health, as higher PHYA levels are correlated with lower symptom rates, which would result in better bone health. On the other hand, the relationship between PHYA and bone health can be moderated by mental disorders (Fig. 1). The interplay between regular PHYA and mental disorders would require an adapted treatment approach for bone health in depressed patients and therefore should be assessed.

Accordingly, the aims of this study are to 1. assess the influence of mental disorders on bone markers and 2. evaluate whether the relationship of physical activity with bone markers is influenced by these mental disorders.

## 2 Methods

### 2.1 Study design and population

A secondary analysis was conducted on data from a sub-set (*n* = 44) of depressed patients recruited at intake in a psychosomatic rehabilitation clinic, stemming from a previous project (DEPREHA (Wippert et al., 2019)). The data used for the current manuscript were collected from 2015 to 2017 as part of a cohort study, for which further follow-up assessments are planned at later time points. The relevance of the relationship between mental disorders and musculoskeletal conditions, including bone disorders, has only recently gained increasing scientific attention and is of particular importance in an aging society such as the German population (Hadji et al., 2024; Hadji et al., 2020). To date, only a limited number of datasets from the German population allow for the simultaneous assessment of mental health and musculoskeletal parameters. The present dataset allows for a better understanding of the complex interactions between mental and musculoskeletal health outcomes.

The recruitment of depressed participants was conducted in Germany, according to the following inclusion criteria: participants aged 18–65 years suffering from a depressive episode (ICD-10 F32.x or F33.x) depression diagnosis, dysthymia (F34.1), or having any other adjustment disorder with an extended depressive reaction (F43.21). Further inclusion criteria consisted of the inability to work for ≥21 days over the past 12 months due one of the diagnoses mentioned above. Exclusion criteria consisted of pregnancy, hormonal therapy (except hormonal contraception), intellectual disabilities (ICD10 F70–89), conformity with other disorders (e.g., endocrine or metabolic disorders), neurological disorders; dementia (ICD-10 F00-F03), psychotropic drug dependence syndrome (ICD-10 F1x.2), schizophrenia (ICD-10 F20), psychotic, stress, and somatoform disorders (F40–49, apart from the disorders mentioned in the inclusion criteria), emotionally unstable personality disorder (ICD-10 F60.3×), as well as other personality and behavioral disorders (F61-F69), acute infections, immune system disorders, unstable remitting addictions and acute drug abuse (excluding nicotine).

All participants in this experiment were informed of the purpose and content of the study both verbally and in written form, and all participants signed the informed consent form. The study was conducted according to the principles of the Declaration of Helsinki. Final ethics approval was obtained on 11.12.2017 from the Ethics Review Board of the University of Potsdam, Germany (number 15/2017).

### 2.2 Measures

#### 2.2.1 Questionnaires

*Sociodemographic data* (age, sex, BMI, etc.) were collected through questionnaires during the pre-assessment of patients at clinic admission by medical and research personnel.

For reasons of clarity, depressive disorders and somatoform disorders were subsumed under the umbrella term *mental disorders* in this study. *Depressive symptoms* were measured with the screening tool the Beck Depression Inventory II (BDI-II) (Wintjen and Petermann, 2010). The BDI-II is a self-reported questionnaire, including 21 questions answered on a four-point Likert scale, which measures the severity of the symptoms of depression among adolescent and adult depressed patients. The internal consistency of the BDI-II questionnaire was estimated as Cronbach's alpha 0.890 (Wippert et al., 2019).

The Symptom Checklist (SCL-90) Revised measured *psychosomatic symptoms* over the past seven days (Franke, 2002). The SCL-90 is composed of 90 items divided in nine dimensions (somatization, obsessive-compulsive, interpersonal sensitivity, depression, anxiety, hostility, phobic anxiety, paranoid ideation, and psychoticism). The questions are answered on a five-point Likert scale ranging from zero (not at all) to four (extremely). The sum of all scale values was used for analysis, with a Cronbach's alpha of 0.896 in the current sample.

Whether patients partook in regular PHYA prior to the study was assessed in the medical pre-assessment. At intake, doctors registered whether patients were physically active or/and participated in sports in their daily life. Based on this information, participants were classified as either ‘physically active’ or ‘physically inactive’.

#### 2.2.2 Bone markers

Participants donated a blood sample of 10 mL, from which serum was extracted for analysis. The blood samples and questionnaires were collected within a 24-h timeframe. Before the blood draw, participants were instructed to fast for 12 h, consuming only water. Additionally, they were advised to avoid unscheduled or heavy medication and to limit their intake of tea and coffee, as well as refrain from intense exercise for 24 h prior to the donation. Participants were also required to follow a specific nutrition protocol (Wippert et al., 2019). The included bone markers consisted of Procollagen type 1 N-propeptide (P1NP), osteocalcin (OC) and crosslaps (CTx). Bone marker levels were analyzed from blood serum samples with electrochemiluminescence immunoassays (ECLIA) from Roche COBAS Elecsys 2010 MODULAR ANALYTICS E170 (REF 12149133 122 for Osteocalcin, REF 03141071 190 for P1NP and REF 11972308 122 for CTx, F. Hoffmann-La Roche, Ltd., Basel, Switzerland) at the Ernst von Bergman Clinic in Potsdam.

### 2.3 Statistical analysis

A P1NP/CTx ratio, representing bone turnover, was calculated from the P1NP and CTx expression values. Differences between the physically active and inactive groups regarding participant characteristics (sociodemographic data, BDI-II and psychosomatic symptoms, and bone marker concentrations) were calculated using the Mann-Whitney *U* test (age, BMI, and bone markers; no normal distribution), independent samples *t*-tests (two-tailed; BDI-II and SCL90; assuming normality) and Chi-square tests (sex, BDI-II severity, recurring depression, antidepressant medication) with a significance level set at *p* < 0.05.

A multiple linear regression (adjusted for age and sex) was conducted to detect the individual influences of BDI-II and SCL-90 scores on P1NP, OC, and CTx (*p* < 0.05). A second multiple linear regression assessed the interaction (moderation) of PHYA with BDI-II (centered) and PHYA with SCL-90 (centered) to detect whether depressive or psychosomatic symptoms interfere with the association of PHYA and bone markers. Finally, the PROCESS SPSS macro (Hayes, 2022) was used to conduct mediator (model four) analysis for depressive and psychosomatic symptoms on the relationship between PHYA and bone markers. The heteroscedasticity consistent covariance matrix (HCCM) HC3 was chosen, as recommended for small samples by Long and Ervin (2000). Bootstrapping was used to detect bootstrap confidence intervals (CI), in which lower and upper CI not surrounding zero indicates significance. All descriptive and inferential statistics were performed in IBM SPSS Statistics version 31.0.

## 3 Results

Participant characteristics, divided for physically active and inactive groups, are provided in Table 1 in mean ± SD, with the *p*-value representing group differences. Mean age was 47.57 ± 9.18 years with 86 % of participants being female, and a BMI of 28.93 ± 6.15 kg/m^2^. No significant differences in sociodemographic factors between the active and inactive group were found.

**Table 1:** Participant characteristics and differences in depressive and psychosomatic symptoms, bone-related and inflammatory parameters between the physically active and inactive groups of the included depressed participants in Germany (2015–2017).

	N	All	N	Physically active	N	Physically inactive	*p*-value
Sex (M/F)	44	6/38	25	4/21	19	2/17	0.60
Age (y)	44	47.6 ± 9.2	25	47.8 ± 10.2	19	47.3 ± 7.8	0.72
BMI (kg/m^2^)	44	28.9 ± 6.2	25	28.0 ± 6.2	19	30.2 ± 6.0	0.25
Depression & Psychosomatic outcomes							
BDI-II	42	25.1 ± 10.3	23	23.0 ± 9.8	19	27.7 ± 10.5	0.15
BDI severity	42	2.9 ± 1.2	23	2.7 ± 1.3	19	3.3 ± 1.1	0.08
SCL90	40	99.6 ± 45.6	22	88.7 ± 48.0	18	113.0 ± 39.8	0.09
Recurring depression	44	27	25	17 (68.0 %)	19	10 (52.6 %)	0.46
Antidepressant medication (y/n)	34	26/8	21	17/4	13	9/4	0.43
Bone markers							
P1NP (ug/L)	40	49.8 ± 18.9	22	50.5 ± 19.0	18	48.9 ± 19.2	0.82
OC (ng/mL)	40	16.7 ± 5.5	22	17.3 ± 5.4	18	16.1 ± 5.6	0.40
CTx (ng/mL)	40	0.3 ± 0.1	22	0.3 ± 0.1	18	0.3 ± 0.1	0.41
P1NP/CTx ratio	40	167.1 ± 55.3	22	161.9 ± 54.5	18	173.5 ± 57.2	0.27

BMI: Body Mass Index; BDI-II: Beck Depression Inventory; SCL90: Symptom Checklist 90; P1NP: Procollagen type 1 N-Propeptide; OC: osteocalcin; CTx: crosslaps; BDI severity was classified as (1) none: 0–8, (2) minimal: 9–13, (3) mild: 14–19, (4) moderate: 20–28, (5) severe: 29–63; Significant results: *p* < 0.05.

### 3.1 Aim 1

The multiple linear regression (Table 2), adjusted for age and sex, showed significant associations of BDI-II with P1NP (B = 0.66; *p* < 0.05) and OC (B = 0.19; *p* < 0.05); SCL-90 with P1NP (B = 0.17; *p* < 0.05).

**Table 2:** Associations (regression coefficient B) of depressive (BDI-II) symptoms and psychosomatic (SCL-90) symptom scores with Procollagen-type-1-N-propeptide, Osteocalcin, and Crosslaps, adjusted for age and sex among the included depressed participants in Germany (2015–2017).

Variables	P1NP		OC		Ctx	
	Coefficient B	*p-*value	Coefficient B	*p-*value	Coefficient B	*p-*value
BDI-II	**0.66**	**0.02**	**0.19**	**0.02**	0.00	0.62
SCL-90	**0.17**	**0.01**	0.04	0.06	0.00	0.81

Significant result is written in bold; PHYA: Physical activity; PHYA: physical activity; BDI-II: Beck Depression Inventory II; SCL-90: Symptom-Checklist-90; P1NP: Procollagen-type-1-N-propeptide; OC: Osteocalcin; CTX: Crosslaps.

**Table 3:** Main and interaction effects (regression coefficient B) of physical activity, depressive (BDI-II) and psychosomatic (SCL-90) symptoms on the bone markers Procollagen-type-1-N-propeptide, Osteocalcin, and Crosslaps among the included depressed participants in Germany (2015–2017).

Variables	Model 1		Model 2		Model 3		Model 4	
	Coefficient B	*p-*value	Coefficient B	*p-*value	Coefficient B	*p-*value	Coefficient B	*p-*value
	**P1NP**							
PHYA	6.74	0.26	6.29	0.27	6.86	0.26	6.14	0.29
BDI-II	**0.65**	**0.03**	0.13	0.07	–	–	–	–
PHYA x BDI-II	–	–	1.07	0.06	–	–	–	–
SCL-90	–	–	–	–	**0.19**	**0.01**	0.03	0.76
PHYA x SCL-90	–	–	–	–	–	–	**0.27**	**0.048**
	**OC**							
PHYA	2.78	0.10	2.68	0.16	2.55	0.16	2.43	0.18
BDI-II	**0.21**	**0.01**	0.08	0.48	–	–	–	–
PHYA x BDI-II	–	–	0.27	0.10	–	–	–	–
SCL-90	–	–	–	–	**0.05**	**0.03**	0.02	0.56
PHYA x SCL-90	–	–	–	–	–	–	0.05	0.28
	**CTx**							
Phya	0.05	0.23	0.05	0.24	0.06	0.22	0.05	0.25
BDI-II	0.00	0.43	−0.00	0.43	–	–	–	–
PHYA x BDI-II	–	–	0.01	0.06	–	–	–	–
SCL-90	–	–	–	–	0.00	0.48	−0.00	0.40
PHYA x SCL-90	–	–	–	–	–	–	0.00	0.10

Significant result is written in bold; PHYA: Physical activity; BDI-II: Beck Depression Inventory II; SCL-90: Symptom-Checklist-90; P1NP: Procollagen-type-1-N-propeptide; OC: Osteocalcin; CTX: Crosslaps.

Model 1: adjusted for BDI-II; Model 2: adjusted for BDI-II (centered) and interaction term (PHYA x BDI-11);

Model 3: adjusted for SCL-90; Model 4: adjusted for SCL-90 (centered) and interaction term (PHYA x SCL-90).

### 3.2 Aim 2

The linear multiple regression (Table 3) showed main and interaction effects of the centered BDI-II and SCL-90 variables on P1NP and OC (Table 3). A significant interaction between PHYA and SCL-90 was observed as a combined influence on P1NP (B = 0.27; *p* < 0.05). The interaction terms of PHYA with BDI-II on P1NP and CTx bordered on significance (*p* = 0.06). No significant mediation effects of BDI-II or SCL-90 on the relationship between PHYA and P1NP, OC and CTx were detected (Appendix A).

## 4 Discussion

The aims of this study were (1) to evaluate whether mental disorders directly influence bone health and (2) assess whether the relationship of PHYA with bone markers is influenced by these mental disorders. This knowledge could give insights to whether mental disorders should be taken into account in designing PHYA therapy offers to improve bone health.

For aim 1, the analysis showed associations of depressive and psychosomatic symptoms with bone markers, indicating an increase in bone marker expression with increased symptom severity. The data for the current analysis stems from participants at intake in an inpatient psychosomatic rehabilitation clinic, indicating that they were suffering from a depressive episode, further supported by the moderate (20–28 points) depressive symptom scores. Therefore, these results indicate that the level of bone marker expression is dependent on mental disorder (symptom) severity among patients suffering from an acute depressive episode, which is in accordance with previous studies showing that increased depressive symptom severity and experiencing a depressive episode are related to higher bone turnover in depressed patients (Petronijević et al., 2008; Herrán et al., 2000).

As it was shown that mental disorders affect bone marker expression, for aim 2, moderation and mediation analyses of mental disorders on the relationship of PHYA and bone markers were conducted. No mediation effects, but small moderating effects of psychosomatic symptoms on the relationship of PHYA with bone markers were found. Similar but non-significant interactions of PHYA with depressive symptoms (BDI-II) (*p* = 0.06) were found, indicating that the association between PHYA and bone metabolism might depend on the level of depressive and psychosomatic symptoms.

The notion that mental disorders moderate relationships between PHYA and other disorders is in accordance with the results of the study of Wippert et al. (2021), who investigated psychosocial moderators and mediators between the relationship of exercise and pain in back pain patients, and found depressive symptomatology to be a moderator between the relationship of exercise and pain, but not a mediator (Wippert et al., 2021). To the authors' knowledge, no other studies have researched the moderation and mediation effects of mental disorders on the relationship between PHYA and bone markers. Li et al. (2024) and Chen et al. (2024) researched the complex triad of depression, bone health and PHYA, but in a different direction (Chen et al., 2024; Li et al., 2024). Li et al. (2024) assessed mediation effects of PHYA (MET minutes/week) on depressive symptoms severity and BMD in a predominantly healthy population (*n* = 7273) (Li et al., 2024); Chen et al. (2024) assessed the prevalence of osteoporosis and depressive symptoms in a nationwide survey (*n* = 11.603; older adults >50 years) and conducted mediation analysis to assess associations between PHYA, osteoporosis and depression (Chen et al., 2024). The researchers from both studies found that *physical activity* mediated the association between depression and bone health (Chen et al., 2024; Li et al., 2024). Even though these studies didn't evaluate a specific depressed sample or the same direction of the association between PHYA and depression and their relationship with bone, as well as the same outcome markers (BMD instead of bone markers), it does strengthen the notion that PHYA and mental disorders have a combined influence on bone health.

The positive influence of PHYA on bone health has been stated in several previous scientific publications (Hejazi et al., 2022; Benedetti et al., 2018). In the current study, physically active depressed patients showed slightly higher bone marker expressions indicating increased bone metabolism, but results were non-significant. For exercise to have a direct positive effect on bone health, some type of (mechanical) loading has to be put on the bone through weight-bearing aerobic exercises (e.g. walking, jogging) or resistance training (Benedetti et al., 2018). The type of PHYA conducted by the sample in this study might have been non-weight-bearing exercises or with a limited duration insufficient to generate bigger effects on bone metabolism.

Bone markers P1NP, OC and CTx were used as markers of bone health in the current study. P1NP and CTx are recommended bone markers for the diagnosis and management of osteoporosis (Bhattoa et al., 2025). Regarding OC, it should be noted that, recently, flaws for the use of OC as a marker of bone turnover have come up. OC seems to be an imperfect marker for high bone turnover in postmenopausal osteoporosis, because of controversial findings among studies so far (Martiniakova et al., 2024). Still, the same review noted the role of OC in various metabolic processes and reviewed OC as a medium involved with treatment response to pharmacological drugs in people with bone-related disorders (Martiniakova et al., 2024). Results from a further review have highlighted that OC responds to exercise training (Mohammad Rahimi et al., 2021), which might make it a suitable marker for PHYA as treatment form among people with bone disorders.

According to the authors' knowledge, this is the first study directly assessing the interaction of somatoform and depressive disorders with physical activity, and its relationship with bone health in depressed patients.

One limitation of the current manuscript is the sample size (*n* = 44), which was small due to the limited availability of PHYA data among the DEPREHA cohort. The moderation and mediation results should therefore be interpreted with caution. The non-significant mediation results from this analysis could either mean that there is indeed no mediation effect, however, the possibility also exists that the study was underpowered and therefore not able to detect a significant effect (Fritz et al., 2015). For the same reason, additional factors, such as recurring depression and antidepressant medication intake were not added as confounding variables for the mediation and moderation analysis, as this would lead to overfitting of the regression models due to the limited sample size available. Yet, the mediation and moderation results provide an indication of the possible moderating influence of mental disorders on physical processes and warrant further research, specifically in larger populations.

Furthermore, BMD measurements through DEXA scans were not included for analysis. BMD was measured in the hip and lumbar spine through DEXA bone densitometry scans in the original study (Wippert et al., 2019). However, the number of patients of whom all relevant data was available, including PHYA, BDI-II and SCL-90 scores, bone marker data and BMD, was limited. The current study therefore utilized solely bone formation and resorption markers as indicators of bone health, where future studies might want to include both BMD as well as bone markers to provide a more complete picture of bone health.

Lastly, PHYA was solely assessed as a binary component (active vs. inactive). Even though 25 participants identified as physically active, their type and intensity of PHYA was not specified, which could have affected the results and limited the effect of PHYA on bone health. Objective measures of PHYA (e.g. fitness trackers, heart rate monitors) were not available, since the data included for the current manuscript consisted of baseline data. However, research concerning fitness trackers as well as PHYA questionnaires have shown limitations, with low accuracy of measuring energy expenditure (Germini et al., 2022) and limited validity of questionnaires (Sember et al., 2020), and would therefore not necessarily provide more valid data.

## 5 Conclusion

Mental disorders affect bone markers and may possibly moderate the relationship between PHYA and bone, which indicates the need to take them into account when prescribing PHYA treatment to improve bone health. With further research, these results could contribute to the effectiveness of treatment, by considering mood disorders, such as depression, when prescribing PHYA therapy programs for the treatment of bone pathologies.

## CRediT authorship contribution statement

**Sanne Houtenbos:** Writing – review & editing, Writing – original draft, Methodology, Formal analysis. **Yangyang He:** Writing – review & editing. **Andrea Block:** Project administration, Data curation. **Pia-Maria Wippert:** Writing – review & editing, Supervision, Project administration, Data curation, Conceptualization.

## Funding sources

This research did not receive any specific grant from funding agencies in the public, commercial, or not-for-profit sectors. The original DEPREHA project was funded by the DRV Berlin-Brandenburg.

## Declaration of competing interest

The authors declare that they have no known competing financial interests or personal relationships that could have appeared to influence the work reported in this paper.

## Data Availability

The datasets used and/or analyzed during the current study are available from the corresponding author upon reasonable request.

## Appendix A Supplementary data

Results Mediation analysis. Supplementary material
