# Supplementary material for: Effects of mental disorders on the relationship between physical activity and bone markers among depressed patients in Germany
Source: Prev Med Rep. 2025 Dec 27;61:103364. doi: 10.1016/j.pmedr.2025.103364 (PMC12808824; doi:10.1016/j.pmedr.2025.103364)
Supplement: Supplementary file 1 — Results Mediation analysis. [file mmc1.docx]

**Appendix A – Mediation analysis**

Table 1: Mediation effects of depressive (BDI-II) and psychosomatic (SCL90) symptoms on the relationship between physical activity and bone markers among depressed patients in Germany.

| Effect | Path | *B* | SE | 95% CI | | *t* | *p* | *R^2^* |  |
| --- | --- | --- | --- | --- | --- | --- | --- | --- | --- |
|  |  |  |  | Lower | Upper |  |  |  |  |
| **BDI-II** (*n* = 38) | | | | | | | |  |  |
| P1NP | | | | | | | |  |  |
| a 🡪 b | PHYA 🡪 BDI-II | -4.17 | 3.50 | -11.27 | 2.94 | -1.19 | 0.24 | 0.04 |  |
| b 🡪 c | BDI-II 🡪 P1NP | 0.65 | 0.34 | -0.04 | 1.35 | 1.91 | 0.06 | 0.14 |  |
| Direct | PHYA 🡪 P1NP | 6.74 | 5.72 | -4.87 | 18.34 | 1.18 | 0.25 | 0.14 |  |
| Total | PHYA🡪 P1NP | 4.02 | 6.27 | -8.70 | 16.73 | 0.64 | 0.53 | 0.01 |  |
| Indirect | Effect | BootSE | | | BootLLCI | | BootULCI | |  |
|  | -2.72 | 2.65 | | | -8.80 | | 1.62 | |  |
| OC | | | | | | | |  |  |
| a 🡪 b | PHYA 🡪 BDI-II | -4.17 | 3.50 | -11.27 | 2.94 | -1.19 | 0.24 | 0.04 |  |
| b 🡪 c | BDI-II 🡪 OC | 0.21 | 0.08 | 0.04 | 0.38 | 2.48 | 0.02 | 0.19 |  |
| Direct | PHYA 🡪 OC | 2.79 | 1.64 | -0.55 | 6.12 | 1.70 | 0.10 | 0.19 |  |
| Total | PHYA 🡪 OC | 1.92 | 1.80 | -1.74 | 5.57 | 1.06 | 0.30 | 0.03 |  |
| Indirect | Effect | BootSE | | | BootLLCI | | BootULCI | |  |
|  | -0.87 | 0.79 | | | -2.63 | | 0.48 | |  |
| CTx | | | | | | | |  |  |
| a 🡪 b | PHYA 🡪 BDI-II | -4.17 | 3.50 | -11.27 | 2.94 | -1.19 | 0.24 | 0.04 |  |
| b 🡪 c | BDI-II 🡪 CTx | 0.00 | 0.00 | -0.00 | 0.01 | 0.78 | 0.44 | 0.05 |  |
| Direct | PHYA 🡪 CTx | 0.05 | 0.04 | -0.03 | 0.13 | 1.25 | 0.22 | 0.05 |  |
| Total | PHYA 🡪 CTx | 0.04 | 0.04 | -0.04 | 0.13 | 1.06 | 0.30 | 0.03 |  |
| Indirect | Effect | BootSE | | | BootLLCI | | BootULCI | |  |
|  | -0.01 | 0.01 | | | -0.03 | | 0.01 | |  |
| **SCL90** (*n* = 36) | | | | | | | |  |  |
| P1NP | | | | | | | |  |  |
| a 🡪 b | PHYA 🡪 SCL90 | -23.11 | 15.01 | -53.62 | 7.40 | -1.54 | 0.13 | 0.07 |  |
| b 🡪 c | SCL90 🡪 P1NP | 0.19 | 0.08 | 0.04 | 0.35 | 2.56 | 0.02 | 0.20 | |
| Direct | PHYA 🡪 P1NP | 6.86 | 5.92 | -5.19 | 18.91 | 1.16 | 0.26 | 0.20 | |
| Total | PHYA 🡪 P1NP | 2.41 | 6.52 | -10.85 | 15.67 | 0.37 | 0.71 | 0.00 | |
| Indirect | Effect | BootSE | | | BootLLCI | | BootULCI | | |
|  | -4.45 | 3.07 | | | -10.94 | | 1.07 | | |
| OC | | | | | | | |  | |
| a 🡪 b | PHYA 🡪 SCL90 | -23.11 | 15.01 | -53.62 | 7.40 | -1.54 | 0.13 | 0.07 | |
| b 🡪 c | SCL90 🡪 OC | 0.05 | 0.02 | 0.01 | 0.09 | 2.33 | 0.03 | 0.15 | |
| Direct | PHYA 🡪 OC | 2.55 | 1.85 | -1.21 | 6.31 | 1.38 | 0.18 | 0.15 | |
| Total | PHYA 🡪 OC | 1.50 | 1.88 | -2.32 | 5.32 | 0.80 | 0.43 | 0.02 | |
| Indirect | Effect | BootSE | | | BootLLCI | | BootULCI | | |
|  | -1.05 | 0.82 | | | -2.97 | | 0.23 | | |
| CTx | | | | | | | |  | |
| a 🡪 b | PHYA 🡪 SCL90 | -23.11 | 15.01 | -53.62 | 7.40 | -1.54 | 0.13 | 0.07 | |
| b 🡪 c | SCL90 🡪 CTx | 0.00 | 0.00 | -0.00 | 0.00 | 0.84 | 0.41 | 0.05 | |
| Direct | PHYA 🡪 CTx | 0.06 | 0.04 | -0.04 | 0.15 | 1.24 | 0.22 | 0.05 | |
| Total | PHYA 🡪 CTx | 0.05 | 0.04 | -0.04 | 0.14 | 1.07 | 0.29 | 0.03 | |
| Indirect | Effect | BootSE | | | BootLLCI | | BootULCI | | |
|  | -0.01 | 0.01 | | | -0.03 | | 0.02 | | |

P1NP = Procollagen type 1 N-Propeptide; OC = osteocalcin; CTx = crosslaps; PHYA = Physical activity; BDI-II = Beck Depression Inventory; SCL-90 = Symptom Checklist 90; *B* = Effect; SE = Standard Error; 95% CI = confidence Intervals; LLCI = lower limit confidence interval; ULCI = upper limit confidence interval; *t=* t-statistic; *p* = significance; *R^2^* = proportion of variance in the dependent variable, which can be explained by the independent variable.
